# Correlation between neutrophil/lymphocyte ratio and cognitive impairment in cerebral small vessel disease patients: A retrospective study

**DOI:** 10.3389/fneur.2022.925218

**Published:** 2022-08-05

**Authors:** Lan Hou, Shuhan Zhang, Dandan Qi, Tongle Jia, Huan Wang, Wei Zhang, Shuyan Wei, Conglong Xue, Pei Wang

**Affiliations:** ^1^Department of Neurology, Baoding No.1 Central Hospital, Baoding, China; ^2^Baoding City Key Laboratory of Neurological Diseases, Baoding, China; ^3^Department of Neurosurgery, Baoding No.1 Central Hospital, Baoding, China; ^4^Department of General Surgery, Baoding No.1 Central Hospital, Baoding, China

**Keywords:** cognitive impairment, Montreal cognitive assessment, Montreal cognitive assessment (MoCA) scale, cerebral small vessel disease (CSVD), neutrophil/lymphocyte ratio (NLR)

## Abstract

**Background and objective:**

The blood neutrophil/lymphocyte ratio (NLR) is an objective and convenient parameter of systemic inflammation. Elevated NLR is associated with an increased risk of mild cognitive impairment (CI) in the elderly. However, few data are available on the impact of the NLR on CI in patients with cerebral small vessel disease (CSVD).

**Methods:**

A total of 66 CSVD subjects with CI and 81 CSVD subjects without CI were evaluated in this study. Clinical, laboratory, radiological, and cognitive parameters were collected. The NLR was obtained with the absolute neutrophil count being divided by the absolute lymphocyte count in fasting blood samples. Logistic regression analysis was performed to evaluate the factors associated with CI. Receiver operating characteristic curves were illustrated to predict factors associated with CI in patients with CSVD.

**Results:**

The NLR of the CI group was significantly higher than that of subjects without CI (2.59 vs. 2.21, *P* = 0.003). In multivariate analysis, NLR was positively correlated to the CI (OR: 1.43, 95% CI: 1.05–1.96, *P* = 0.024). It was suggested that the optimum NLR cutoff point for CI was 1.89 with 69.7% sensitivity and 59.3% specificity. Subjects with NLR ≥ 1.89 showed higher possibilities of CI compared to those with NLR < 1.89 (OR: 3.38, 95% CI: 1.62–7.07).

**Conclusions:**

Correlations were found between NLR and CI. Patients with CSVD who have higher NLR might have an increased risk of CI.

## Introduction

Cerebral small vessel disease (CSVD) is a latent, chronic and progressive disorder of small arterioles, capillaries, or veins, usually 50–400μm in diameter, penetrating and supplying the subcortical region, the white matter, and deep structures of gray matter. It is an increasingly prevalent age-related condition affecting 5% of participants aged over 50 and almost everyone in the age of 90 (1). As the use of magnetic resonance imaging (MRI) is increasing, more and more cases of CSVD are detected.

Cerebral small vessel disease is a diverse spectrum of neuropathological and neuroimaging processes. Cognitive impairment (CI) is one of the major manifestations in patients with CSVD (2). Since most patients with CSVD are initially asymptomatic, it is crucial for early detection of CI. However, a professional neuropsychological evaluation is not available in some rural areas. Therefore, efforts should be made to find nonexpensive and easy tests that can help to identify candidates for cognitive assessment.

The pathogenesis of CSVD remains unclear. Accumulating evidence demonstrates inflammation as a key role in vascular damage (3). Systemic inflammation can trigger a pro-inflammatory environment in the central nervous system, which thereafter potentially aggravates the molecular cascades in cerebral vessel disease (4).

The neutrophil/lymphocyte ratio (NLR) is defined as the ratio of neutrophils to lymphocytes. It is an objective and convenient parameter of systemic inflammation (5), reflecting the balance and imbalance between neutrophils and lymphocytes in peripheral blood. A community-based study focusing on elderly adults with age over 65 years demonstrated that NLR was associated with the risk of CI (6). However, the role of NLR in patients with the diagnosis of CSVD is unclear.

Therefore, we hypothesized that patients with CSVD with increased inflammation may be more prone to developing CI. We designed a study and aimed to analyze whether NLR was associated with CI in patients with CSVD.

## Materials and methods

### Study population

**Figure 1:**
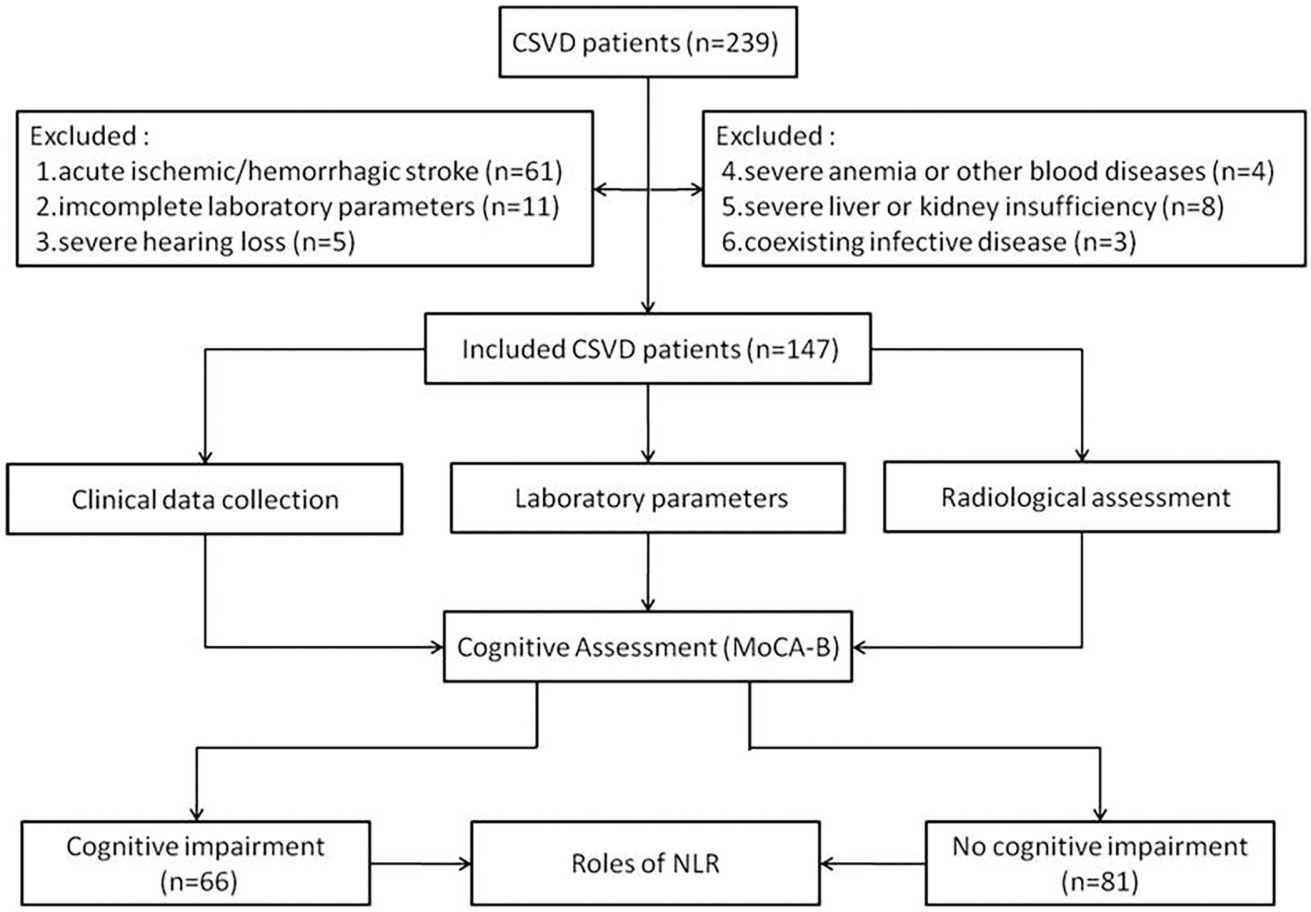
The study flow chart.

This was a hospital-based, retrospective study and was performed from September to December 2019 and from October 2020 to May 2021, as seen in Figure 1. A study population of 239 CSVD inpatients who underwent cranial MRI examination, including fluid-attenuated inversion recovery (FLAIR) and susceptibility weighted imaging (SWI) sequence, at the neurology department of Baoding No.1 Central Hospital, Boading, China was included. Of these, 92 individuals were excluded because of acute ischemic or hemorrhagic stroke (*n* = 61), incomplete laboratory parameters (*n* = 11), severe anemia or other blood diseases (*n* = 4), severe liver or kidney insufficiency (*n* = 8), coexisting infective disease (*n* = 3), and severe hearing loss that cannot coordinate with the cognitive evaluation (*n* = 5). All the participants were examined by neurologists, psychiatrists, and radiological investigators. CSVD was diagnosed according to the CSVD criteria of the Chinese guidelines (7). The time from the diagnosis of CSVD and the blood tests was within 3 days.

### Radiological assessment

Using Philips 1.5T magnetic resonance scanner, T1 weighted image, T2 weighted image (T2WI), FLAIR, and SWI were performed. The diagnostic criteria of CSVD included white matter hyperintensity (WMH), enlarged perivascular spaces (ePVS), lacunar infarcts (LI), and cerebral microbleeds (CMB) indicated by brain MRI examination (8). WMH was a signal abnormality of different sizes in the white matter shown as hyperintensity on T2WI and FLAIR without cavitation. ePVS were demonstrated as round, oval, or linear lesions with clear boundaries, about <3 mm in diameter. It was filled up with cerebrospinal fluid (CSF) and therefore followed a similar signal intensity to CSF. LI was defined as a round or ovoid fluid-filled cavity (3–15 mm diameter) in the territory supplied by deep perforating arteries. CMB was detected as small round or oval low signals on the SWI sequence (generally 2–5 mm in diameter). Image interpretation was accomplished by one neurologist and two radiologists who were blinded to any clinical information.

### Cognitive assessment

Neuropsychological tests were administered by a single examiner who was blinded to the study design. Because of the high sensitivity and specificity for detecting CI (9), we used the Montreal cognitive assessment (MoCA) scale to evaluate the cognitive function of all participants. The MoCA-Basic (MoCA-B) facilitated the detection of CI in illiterate and low-educated individuals (10). It assessed cognitive domains including modified trail-making test part B (0–1 points), fruit fluency (0–2 points), orientation (0–6 points), problem-solving task (0–3 points), similarity (0–3 points), delayed recall (0–5 points), superimposed object recognition (0–3 points), animal naming (0–4 points), and attention (0–3 points) with a maximum score of 30 points. The thresholds for CI were as follows: 19 for individuals with an education of no more than 6 years, 22 for individuals with an education of 7 to 12 years, and 24 for individuals with an education of more than 12 years. Participants were categorized into CI and non-CI (NCI) groups according to the MoCA-B assessment.

### Collection of clinical and laboratory parameters

Baseline characteristics including age, sex, and body mass index (BMI) were collected. Vascular risk factors evaluated included a history of hypertension, coronary artery disease (CAD), diabetes mellitus, smoking, and alcohol intake. Blood samples from the antecubital vein in all subjects after 6-to-8-h overnight fasting were collected between 5:00 am and 7:00 am and then stored in calcium ethylene diamine tetraacetic acid tubes. Blood cell count analysis including neutrophil, lymphocyte, and monocyte counts was analyzed using an auto-analyzer (XN-9000, Japan) in our hospital. The NLR was calculated by the absolute neutrophil count being divided by the absolute lymphocyte count. All laboratory examinations, including glucose, total cholesterol, triglyceride, low-density lipoprotein (LDL) cholesterol, high-density lipoprotein (HDL) cholesterol, homocysteine, urate nitrogen, creatinine, and uric acid levels, were conducted at the same time.

### Statistical analysis

Statistical processing was applied using the SPSS 25.0 software package. Measurement data were expressed as mean ± standard deviation, followed by the homogeneity of variance test, the *t*-test for comparing data between two groups, and the Mann-Whitney U test for two groups. The adoption rate of count data was calculated, and the chi-square test was used for comparison between groups. Logistic regression analysis was performed to evaluate the factors associated with the CI. Receiver operating characteristic (ROC) curves were illustrated to predict the factors associated with CI in patients with CSVD. Comparison of data between groups was considered statistically significant for a *p*-value < 0.05.

## Results

A total of 147 individuals with CSVD were enrolled in this study. The CI group included 66 subjects and the NCI group included 81 subjects. Details are demonstrated in Table 1. The median age of participants in the CI group was 64.5 years (58.75–70 years), and 57.5% of the participants (*n* = 38) were men with an average BMI of 25.12 ± 3.22. Vascular risk factors included history of hypertension (*n* = 47, 60%), diabetes mellitus (*n* = 22, 28.3%), CAD (*n* = 14, 26.4%), smoking (*n* = 21, 36.4%), and drinking (*n* = 18, 24.5%). In the NCI group, the median age of participants was 61.2 years (54–68.5 years), and 43.2% of the participants (*n* = 35) were men with an average BMI of 24.94 ± 3.26. History of hypertension (*n* = 55, 74.8%), diabetes mellitus (*n* = 20, 28.4%), CAD (*n* = 22, 23.1%), smoking (*n* = 24, 32.6%), and drinking (*n* = 22, 28.4%) were shown in the NCI group. Between the two groups, no significant differences were found in total cholesterol, triglyceride, LDL cholesterol, HDL cholesterol, homocysteine, urate nitrogen, and uric acid levels. Although there was no statistical difference in the counts of neutrophils and lymphocytes between the two groups, patients with CI had statistically elevated NLR levels compared with patients without CI ([2.59 (1.73, 3.09)] vs. [2.21 (1.39, 2.53)], *P* = 0.003). Besides, higher fasting glucose (*P* = 0.008) and creatinine (*P* = 0.046) levels were statistically significant in the two groups.

**Table 1:** Clinical characteristics of participants according to cognitive assessment.

	**CI group (*n =* 66)**	**NCI group (*n =* 81)**	***F*/*H*/χ2**	***P***
Men, *n* (%)	38 (57.5)	35 (43.2)	3.002	0.083
Age, years	64.50 (58.75, 70.00)	61.20 (54.00, 68.50)	−1.920	0.055
BMI, kg/m^2^	25.12 ± 3.22	24.94 ± 3.26	0.467	0.495
Hypertension, *n* (%)	47 (60.0)	55 (74.8)	0.188	0.665
Diabetes mellitus, *n* (%)	22 (28.3)	20 (28.4)	1.331	0.249
CAD, *n* (%)	14 (26.4)	22 (23.1)	0.696	0.404
Current smoking, *n* (%)	21 (26.4)	24 (32.6)	0.082	0.775
Current drinking, *n* (%)	18 (24.5)	22 (28.4)	0.000	0.988
Neutrophil count, (×10^9^/L)	4.21 ± 1.40	3.50 ± 1.05	3.614	0.059
Lymphocyte count, (×10^9^/L)	1.80 ± 0.66	1.79 ± 0.56	1.761	0.187
NLR	2.59 (1.73,3.09)	2.21 (1.39,2.53)	−2.954	0.003*
Fasting glucose, (mmol/L)	6.66 (5.06,7.26)	5.55 (4.92,5.91)	−2.666	0.008*
Total cholesterol, (mmol/L)	4.39 ± 0.96	4.61 ± 1.13	2.125	0.147
Triglycerides, (mmol/L)	1.49 (1.08,1.80)	1.37 (0.87,1.65)	−1.858	0.063
HDL cholesterol, (mg/dL)	1.08 ± 0.25	1.19 ± 0.29	1.009	0.317
LDL cholesterol, (mg/dL)	2.65 ± 0.57	2.75 ± 0.84	1.768	0.186
Homocysteine, (umol/L)	18.11 (11.34, 19.77)	14.85 (9.80, 17.11)	−1.363	0.173
Urate nitrogen, (mmol/L)	5.77 (4.30,6.01)	5.10 (4.03,5.94)	−0.213	0.225
Creatinine, (umol/L)	77.04 (57.36, 76.80)	63.90 (32.27, 74.85)	−1.996	0.046*
Uric acid, (umol/L)	328.1 (241.98, 398.50)	301.60 (249.20, 356.30)	−1.643	0.100
MoCA	15.25 ± 5.66	25.81 ± 2.70	−14.829	0.000*

CI, cognitive impairment; NCI, no cognitive impairment; BMI, body mass index; CAD, coronary artery disease; NLR, neutrophil/lymphocyte ratio; HDL, high-density lipoprotein; LDL, low-density lipoprotein.

* *P* < 0.05.

Next, we conducted logistic regression analyses between the CI and NCI groups to determine whether NLR was an independent factor associated with the CI (Table 2). The regression model included the significant variables: fasting glucose, creatinine, and NLR. It was demonstrated that NLR (OR: 1.43, 95% CI: 1.05–1.96, *P* < 0.05) and fasting glucose (OR: 1.31, 95% CI: 1.06–1.61, *P* < 0.05) were significantly associated with higher possibilities of the occurrence of CI, while creatinine was not an independent factor associated with CI (OR: 1.02, 95% CI:0.09–1.04, *P* = 0.153).

**Table 2:** Results of multiple logistic regression analysis of the possible correlates for CI.

	**ß**	**SE**	**Wald χ2**	***P***	**OR**	**95%CI**
NLR	0.395	0.159	5.061	0.024	1.43	1.05–1.96
Fasting glucose	0.267	0.105	6.421	0.011	1.31	1.06–1.61
Creatinine	0.016	0.011	2.041	0.153	1.02	0.09–1.04

CI, cognitive impairment; NLR, neutrophil/lymphocyte ratio.

**Figure 2:**
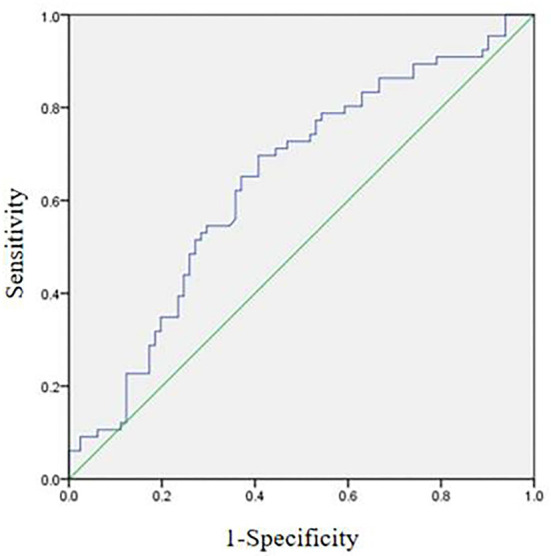
Receiver operating characteristic curve analysis of patients with CI.

Receiver operating characteristic curve analysis was performed to measure the power of the NLR to predict the development of CI and to determine a cutoff (Table 3). It indicated that the optimum NLR cutoff point for CI was 1.89 with 69.7% sensitivity and 59.3% specificity (Figure 2). The area under the curve was found to be 0.642 and yielded a significant result (*P* = 0.003). Meanwhile, the optimum cutoff point of fasting glucose for CI was 5.21 with 71.2% sensitivity and 58.2% specificity. The area under the curve was found to be 0.628 and also yielded a significant result (*P* = 0.008).

**Table 3:** Results of receiver operating characteristic analysis of NLR and other markers in CI.

	**AUC**	**Cutoff**	**Sensitivity (%)**	**Specificity (%)**	**Youden**	**95% CI**
NLR	0.642	1.89	69.7	59.3	0.290	0.55–0.73
Fasting glucose	0.628	5.21	71.2	58.2	0.231	0.54–0.72

CI, cognitive impairment; NLR, neutrophil/lymphocyte ratio.

Table 4 displayed the odds ratios of CI according to the NLR cutoff value of 1.89. Before and after adjustment for other factors, including age, BMI, hypertension, CAD, and diabetes mellitus, subjects with NLR ≥1.89 showed higher risk relative to subjects with NLR <1.89 (unadjusted OR of 3.02, adjusted OR of 3.38).

**Table 4:** Odds ratios of CI according to NLR cutoff before and after adjustment.

**NLR**	**Number of subjects**	**No. of CI (%)**	**Basic model, OR (95% CI)**	**Final model^*a*^,** **OR (95% CI)**
<1.89	66	20 (30.3)	1.000	1.000
≥1.89	81	46 (56.8)	3.02 (1.52–6.00)	3.38 (1.62–7.07)

CI, cognitive impairment; NLR, neutrophil/lymphocyte ratio; OR, odds ratio; 95% CI, 95% confidence interval;

a adjusted for age, BMI, hypertension, CAD, and diabetes mellitus.

## Discussion

In this study, we focused on the association between blood NLR and the risk of CI in patients with CSVD. According to our results, the patients with CI showed a relatively high burden of NLR values in comparison with patients without CI. The NLR was significantly positively correlated with the presence of CI. In the CSVD patients with NLR ≥1.89, the risk of CI was three times higher than that in patients with NLR <1.89. This might offer a meaningful way to estimate CI in patients with CSVD.

It was reported that the NLR was independently associated with an increased risk of CI in elderly individuals (6, 11) and in patients with acute ischemic stroke (4). Elevated NLR was also detected in people with mild CI and early Alzheimer's disease compared with subjective cognitive decline and control groups (12). Our results were in accordance with prior studies relating to NLR and CI. The NLR was associated with CI in patients with CSVD, whereas the underlying mechanism was unclear.

Inflammation is increasingly implicated as a risk factor for CSVD (13). Inflammation not only acts through the immune system but also interacts with traditional cerebrovascular risk factors to exacerbate its harmful effects (14). Inflammation is also believed to play an important role in CI related to Alzheimer's disease (15) or vascular CI (16) pathogenesis. Markers of inflammation are classified into systemic inflammation and vascular inflammation. Systematic reviews explored the relationship between markers of inflammation and CSVD. Although cross-sectional findings on systemic inflammation (e.g., C-reactive protein, interleukin-6, fibrinogen) were controversial, longitudinal investigations demonstrated that elevated levels of systemic inflammatory markers predicted subsequent CSVD severity and progression (13). In a prospective cohort of older participants, faster declines in C reactive protein (CRP) over time were related to indicators of white matter health (17). In a community-based study of 1,532 participants, the individuals whose CRP levels transitioned from low to high during midlife demonstrated the greatest white matter hyperintensity volume and the poorest white matter microstructural integrity (18). Recently, NLR is emerging as an easily accessible indicator of systemic inflammatory status. Besides, it is more stable for measurement and less affected by conditions that could change the individual cell counts (19). Compared with conventional inflammatory markers summarized in a review (13), there are only a few studies on the relationship between NLR and CSVD (20–24).

Neutrophils are known as a pro-inflammatory indicators. Underlying cerebral microstructural changes (i.e., endothelial dysfunction and breakdown of the BBB) have been observed in CSVD. During atherogenesis or ischemic stroke, neutrophils can aggravate the damage through increased release of reactive oxygen species, which increases endothelial dysfunction and permeability (25). Evidence suggests that peripheral neutrophil activation, indicated by blood concentrations of neutrophil gelatinase-associated lipocalin and myeloperoxidase, may be a pathological feature of CI (26). Lymphocyte also plays a dominant role in chronic inflammation (27). Contrary to neutrophils, lymphocytes are defined as healing promotors by secreting interleukin-10 (28). They express interleukin-10 to the ischemic and reperfused areas and may therefore play an important role in healing. The study has demonstrated that some forms of T cells which recognize myelin-associated self-antigens can exert a neuroprotective effect (29). Higher NLR means enhanced innate immunity and/or attenuated adaptive immunity (30), which might reflect the imbalance of the immune system.

Elderly Chinese subjects with NLR ≥ 2.07 showed a higher risk of mild CI compared to those with NLR <2.07 (6). The discriminative factor for post-stroke CI at 3 months after stroke was NLR ≥ 3.80 in patients with acute ischemic stroke (4). However, the cutoff value of NLR in this study is 1.89. It reflects that subtle changes in the immune microenvironment might be observed in CSVD patients with CI.

In this study, we focus on CI in patients with CSVD. For the first time, correlations were found between NLR and CI in patients with CSVD. However, there are several limitations to this study. First, it was a retrospective cross-sectional study; therefore, selection bias should be considered. Besides, an averaged NLR over a period of time might be more suitable for the evaluation of chronic inflammation. Since the cognitive status and NLR might change over time, patients will be followed up in the future. Additional longitudinal studies are necessary to clarify the causal relationship between NLR and CI in patients with CSVD. Second, other conventional inflammatory markers, including erythrocyte sedimentation rate or CRP, were not included in this study. Considering that all patients underwent complete blood count analysis on admission, this might provide more unbiased information. Besides, the number of participants in the study is small. Finally, all the participants were Chinese, so our results may not be extended to other nationalities.

## Conclusions

In conclusion, our study demonstrated that the NLR of the patients with CI was higher than that of CSVD patients without CI. The NLR was significantly associated with higher possibilities of the occurrence of CI. Further large-scale international prospective studies are in need to establish a causal relationship between NLR and CI in CSVD. If positive, patients with higher NLR would be suggested to cognitive screening for early detection of CI.

## Data availability statement

The raw data supporting the conclusions of this article will be made available by the authors, without undue reservation.

## Ethics statement

The studies involving human participants were reviewed and approved by Ethics Committee of Clinical Research of the Baoding No.1 Central Hospital. Written informed consent for participation was not required for this study in accordance with the national legislation and the institutional requirements.

## Author contributions

LH and SZ: design, drafting of manuscript, and acquisition of data. DQ, TJ, and HW: acquisition of data, statistical analyses, and revision of manuscript. WZ, CX, and SW: conception, interpretation of data, and revision of manuscript. PW: conception, design, and final approval of manuscript. All authors contributed to the article and approved the submitted version.

## Conflict of interest

The authors declare that the research was conducted in the absence of any commercial or financial relationships that could be construed as a potential conflict of interest.

## Publisher's note

All claims expressed in this article are solely those of the authors and do not necessarily represent those of their affiliated organizations, or those of the publisher, the editors and the reviewers. Any product that may be evaluated in this article, or claim that may be made by its manufacturer, is not guaranteed or endorsed by the publisher.
